# Our Journal

**Published:** 1852-01

**Authors:** 


### THE MEDICAL EXAMINER.

PHILADELPHIA, JANUARY, 1852.

With this number we enter upon the fifteenth year of our Journal-existence. In tendering our respectful acknowledgements to the profession for the steady and constantly increasing support which has been extended to the Examiner, we take the occasion to renew the pledge of our best exertions to render it worthy of support. Notwithstanding the growth of valuable journals in every quarter, we have the gratification to find that our columns continue the accepted organ of a large body of the most respectable and intelligent members of the profession of all sections of our country. With our department of '*Original Communications*,' we shall still connect, as heretofore, the publication of clinical and other lectures and hospital reports. In our '*Bibliographical*' department we shall endeavor to present succinct notices of all the new publications on medicine and the collateral sciences. The '*Record*' will be, as formerly, carefully collated from the various European and American journals, with proper attention to desirable *condensation* and *abridgement*. In the '*Editorial*' and '*News*' departments, we hope that our readers will find no abatement of interest, or relaxation of independence of tone and of devotion to the great cause of medical progress. We feel deeply the responsibility that devolves upon us from the editorial control of one of the oldest medical journals of the country; and we trust that we shall maintain its now well-established character.

